# Self‐Assembled Supramolecular Hybrid Hydrogel Beads Loaded with Silver Nanoparticles for Antimicrobial Applications

**DOI:** 10.1002/chem.202001349

**Published:** 2020-06-11

**Authors:** Carmen C. Piras, Clare S. Mahon, David K. Smith

**Affiliations:** ^1^ Department of Chemistry University of York Heslington York YO10 5DD UK

**Keywords:** antimicrobial, gel, nanoparticle, self-assembly, supramolecular chemistry

## Abstract

This Full Paper reports the formation of silver (Ag) NPs within spatially resolved two‐component hydrogel beads, which combine a low‐molecular‐weight gelator (LMWG) DBS‐CONHNH_2_ and a polymer gelator (PG) calcium alginate. The AgNPs are formed through in situ reduction of Ag^I^, with the resulting nanoparticle‐loaded gels being characterised in detail. The antibacterial activity of the nanocomposite gel beads was tested against two drug‐resistant bacterial strains, often associated with hospital‐acquired infections: vancomycin‐resistant *Enterococcus faecium* (VRE) and *Pseudomonas aeruginosa* (PA14), and the AgNP‐loaded gels showed good antimicrobial properties against both types of bacteria. It is suggested that the gel bead format of these AgNP‐loaded hybrid hydrogels makes them promising versatile materials for potential applications in orthopaedics or wound healing.

## Graphical abstract

**Hybrid gel beads** loaded with silver nanoparticles show antimicrobial activity against drug‐resistant bacteria. Each component plays a unique role: the calcium alginate polymer gelator provides mechanical strength and enables shaping into beads, and the dibenzylidenesorbitol low‐molecular‐weight gelator gives control over AgNP formation within the beads.

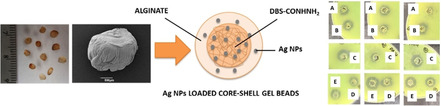

## Introduction

Hydrogels self‐assembled from low‐molecular‐weight gelators (LMWGs) have seen intensive development in recent years for different types of biological applications including cell culture,^1^ tissue engineering,^2^ drug delivery,^3^ antimicrobial therapy^4^ and wound healing.^5^ These materials are obtained from the self‐assembly of small molecules in water, which interact through non‐covalent interactions to form complex entangled fibrillar networks.^6^ One of the most interesting properties of such gel networks is their tunability and responsiveness to external stimuli such as pH, temperature and light.^7^ However, since such gels are assembled via non‐covalent interactions, they are often mechanically very weak, and can be difficult to manipulate. When designing high‐tech programmable materials, spatio‐temporal control over gelation to obtain spatially resolved, shaped gels is a highly desirable feature that can be achieved using different technologies including moulding, 3D printing and photopatterning.^8^ The combination of a LMWG with a polymer gelator (PG) to give a multi‐component hybrid gel combines the responsiveness of the LMWG with the overall mechanical performance of the PG, potentially allowing the gels to be moulded into desired shapes.^9^ This can enable tailoring of gels for specific applications.

We recently developed a highly functional, synthetically‐simple LMWG 1,3:2:4‐di(4‐acylhydrazide)‐benzylidenesorbitol (DBS‐CONHNH_2_),^10^ demonstrating that this hydrogelator has potential applications ranging from environmental remediation to drug formulation and tissue engineering.^11^ This LMWG can achieve efficient reduction of precious metals, inducing the in situ formation of metal nanoparticles (NPs).11a, 12 By combining this LMWG with the PG calcium alginate, we very recently reported that self‐assembly of the LMWG could be spatially controlled by the PG to give core–shell gel beads, in which the PG shell imposes a shape on a self‐assembled LMWG core.^13^ The self‐assembled DBS‐CONHNH_2_ gel core retained its ability to generate palladium nanoparticles, and the resulting gel beads could catalyse cross‐coupling reactions and operated as an efficient recyclable reaction dosage system.

**Figure 1:**
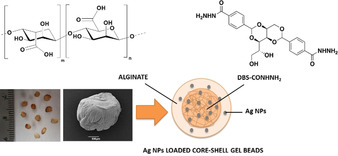
Structures of alginic acid and DBS‐CONHNH_2_, photograph (left) and SEM image (right) of hybrid DBS‐CONHNH_2_/alginate gel beads incorporating Ag NPs and schematic diagram of an AgNP‐loaded core–shell gel bead.

To extend the potential of our innovative gel bead platform into the field of biomedicine, this paper reports the in situ formation of silver (Ag) NPs in the two‐component DBS‐CONHNH_2_/alginate system (Figure 1). AgNPs are known to prevent biofilm formation and have antimicrobial properties against multiple drug‐resistant bacterial strains.^14^ Implant‐associated infections and biofilm formation on implant surfaces are common complications after orthopaedic surgery.^15^ The application of AgNPs on orthopaedic implants to avoid infections and prevent biofilm formation has recently gained some interest as an alternative to the administration of antibiotics.^16^ Furthermore, Ag can have an osteogenic effect on stem cells, promoting bone formation.^17^ Nanomaterials with antibacterial activity are an important and burgeoning field of activity.^18^ In this regard, there has been some interest in the development of supramolecular gels with embedded silver nanoparticles.^19^ In general, these have mostly been unshaped materials formed in vials. Nonetheless, there has been some investigation of the antimicrobial potential of such gels.^20^ We reasoned that the development of AgNP‐loaded shapable biomaterials as bone fillers may ultimately be able to effectively help bone regeneration whilst preventing infections. Such materials could be easily handled and administered to patients as a result of the gel bead format. Indeed, bead‐shaped constructs with antibacterial activity, composed of non‐degradable polymers or degradable biomaterials such as calcium sulfate, are already in clinical use for treatment of bone and wound infections^21^—our systems based on biocompatible individual components11c, 22 and being potentially degradable soft materials, could have significant potential in this setting. As such, in this paper, we make the first report of shaped antimicrobial AgNP‐loaded gel beads based on LMWG technology.

## Results and Discussion

Sodium alginate and DBS‐CONHNH_2_ were combined as previously described by us,^13^ to give a multicomponent gel. Sodium alginate (low viscosity) is commercially available and was purchased and used as supplied. DBS‐CONHNH_2_ was synthesized in good yield by applying our previously reported method.^10^, ^23^ The two networks could be spatially organised in two different ways, depending on the preparation method.

1. Extended interpenetrating networks were obtained by combining an aqueous suspension (1 mL final volume) of DBS‐CONHNH_2_ (0.3 % wt/vol) with alginate (0.5 % wt/vol). Heating ensured the complete dissolution of the LMWG and gelation was triggered on cooling. Once the DBS‐CONHNH_2_ gel had formed, an aqueous solution of CaCl_2_ (5 % wt/vol; 1 mL) was added on top of the gel to cross‐link the alginate by diffusion of calcium ions.
2. Core–shell structured gel beads were prepared by combining the same quantities of DBS‐CONHNH_2_ and alginate, but after heating, the resulting hot solution was added dropwise (20 μL drops) to an aqueous solution of CaCl_2_ (5 % wt/vol) to give small gel beads (ca. 3 mm diameter) on cross‐linking of the alginate with self‐assembly of the DBS‐CONHNH_2_ occurring simultaneously as the system cooled.

Single‐component gels based on DBS‐CONHNH_2_ (in vials) or calcium alginate (in vials and as beads) were also prepared as control materials.

**Figure 2:**
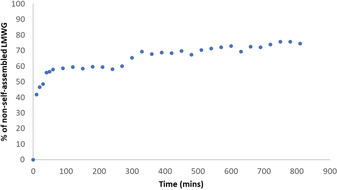
Overnight ^1^H NMR study at 90 °C, showing the percentage of DBS‐CONHNH_2_ that is mobile and hence in a “liquid‐like” non‐self‐assembled state within the gel beads.

These two‐component gels were previously characterised by us in detail,^13^ however as additional characterisation, we were interested here in determining whether DBS‐CONHNH_2_ retained the responsive properties characteristic of a supramolecular gel within our beads. We reasoned that on increasing the ambient temperature, the LMWG network within the core of the gel beads should disassemble within the calcium alginate shell. This would clearly demonstrate the dual nature of these LMWG/PG hybrids. We therefore performed a ^1^H NMR spectroscopy study; this method is a powerful way of determining whether individual gelator molecules are self‐assembled into “solid‐like” nanofibres (and hence invisible by NMR) or are mobile within the “liquid‐like” phase (in which case ^1^H NMR resonances are observed).^24^ We prepared 10 gel beads in D_2_O and transferred them to a NMR tube with 0.5 mL of D_2_O and 2 μL of DMSO as the internal standard. A ^1^H NMR spectrum was immediately recorded to confirm that the DBS‐CONHNH_2_ incorporated into the gel beads was in its self‐assembled state. We then heated the sample at 90 °C, and recorded the ^1^H NMR spectrum at regular time intervals (Figure S1). The percentage of mobile DBS‐CONHNH_2_ within the gel beads was thus calculated (Figure 2, Table S1). After only 10 min at 90 °C, ca. 40 % of the DBS‐CONHNH_2_ became mobile. This increased over time to reach ca. 75 % after 8 h. It is therefore clear that although the calcium alginate provides these gel beads with their shape, the self‐assembled DBS‐CONHNH_2_ retains its thermally responsive characteristics. This clearly indicates how using LMWG/PG hybrid gels can endow hybrid hydrogels with orthogonal properties and suggests that DBS‐CONHNH_2_ within the gel bead core can behave in a similar way to any other sample of this LMWG.

**Figure 3:**
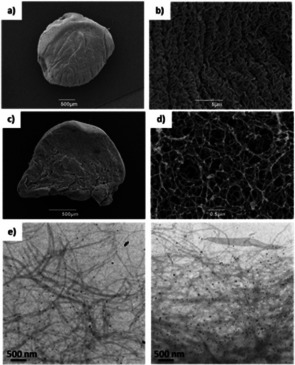
SEM and TEM images of DBS‐CONHNH_2_/alginate gel beads incorporating AgNPs and TEM images of DBS‐CONHNH_2_ gel incorporating AgNPs. a) SEM image of a DBS‐CONHNH_2_/alginate gel bead (scale bar 500 μm); b) SEM image of DBS‐CONHNH_2_/alginate gel bead surface (scale bar 5 μm); c) SEM image of a cross section of a DBS‐CONHNH_2_/alginate gel bead (scale bar 500 μm); d) SEM image of a cross‐section of a DBS‐CONHNH_2_/alginate gel bead (scale bar 0.5 μm). e) TEM images of DBS‐CONHNH_2_/alginate gel bead cross‐section (scale bars 500 nm). SEM images of the gels with no Ag NPs are reported in the Supporting Information (Figures S8 and S9).

**Figure 4:**
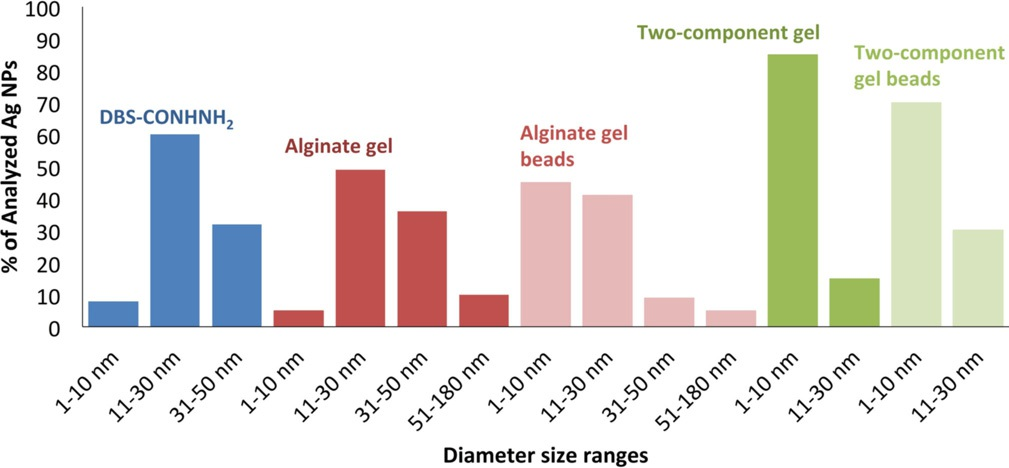
Ag NPs diameter size ranges in DBS‐CONHNH_2_, alginate gels and gel beads and DBS‐CONHNH_2_/alginate gels and gel beads.

Having demonstrated the orthogonal nature of the LMWG/PG networks, we then went on to load these gels with silver. To trigger the in situ formation of AgNPs, gel samples were simply left to interact with AgNO_3_ (1 or 3 mL of a 10 mm solution) for 3 days. The DBS‐CONHNH_2_ gel and the DBS‐CONHNH_2_/alginate hybrid gels changed colour to orange/brown as the Ag^+^ ions diffused into them, clearly indicating the incorporation and in situ reduction of Ag^I^ to Ag^0^ (Figures S2 and S3). After 3 days, the supernatant was removed and the gels were washed with water multiple times. Transmission electron microscopy (TEM) confirmed the formation of AgNPs, with the majority of the diameters being in the range of 1–10 nm for the DBS‐CONHNH_2_/alginate hybrid gels and gel beads (Figure 3, Figure S4). Control experiments with a DBS‐CONHNH_2_ gel indicated AgNPs with the majority of the diameters in the range of 10–30 nm (Figure S5). We observed that calcium alginate gels and gel beads could also incorporate Ag. In this case, however, the Ag‐loaded alginate gel was colourless suggesting either the Ag is present in the +1 oxidation state, or that reduction to Ag^0^ NPs is less effective than in the case where DBS‐CONHNH_2_ is present (where the gels become intensely coloured). We did observe nanoparticles in the calcium alginate gels by TEM (1–50 nm diameter), but they were irregular and tended to aggregate into significantly larger particles with a diameter >150 nm (Figures S6 and S7). It is known that AgNPs can be formed in alginate gels, normally facilitated by heating, manipulation of pH or exposure to UV light.^25^ Indeed, silver‐loaded alginate gels have been of interest in biomedical applications for their anti‐microbial properties.^26^ Overall, in our hybrid hydrogels, where DBS‐CONHNH_2_ is present, silver uptake was significantly better controlled in terms of nanoparticle size (Figure 4). In summary therefore, AgNPs can be incorporated into the gels—clearly facilitated by the presence of DBS‐CONHNH_2_.

The incorporation of Ag into the gels was quantitatively evaluated by analysis of the remaining supernatant solution. A precipitation titration was performed in the presence of NaCl using K_2_CrO_4_ as an indicator. The volume of supernatant required as a titrant, was used to calculate the concentration of residual Ag in the supernatant (i.e., the Ag that was not incorporated into the gel) and, from this, the amount of Ag loaded into the gel could be determined (Table S2). When the gels were loaded with a relatively low amount of AgNO_3_ (10 μmol), the DBS‐CONHNH_2_ gel displayed a better uptake capacity (>9 μmol of Ag mL^−1^ of gel) than the extended interpenetrating hybrid networks (7 μmol of Ag mL^−1^ of gel) or the hybrid gel beads (6.5 μmol of Ag mL^−1^ of gel). We suggest that this is because interactions between DBS‐CONHNH_2_ and alginate networks in the multicomponent gel (as previously described^13^) somewhat limit the ability of the acylhydrazide to reduce Ag^I^ to Ag^0^. However, on exposure to higher concentrations of silver (30 μmol of AgNO_3_), we were able to maximise the uptake of silver. For the interpenetrating network hybrid gel, we could achieve a maximum of 15 μmol of Ag mL^−1^ of gel. When the gel was prepared as hybrid gel beads, the maximum uptake was slightly higher (18 μmol of Ag mL^−1^ of gel) possibly due to the larger surface area of the beads. The hybrid gel contains 6.3 μmol of DBS‐CONHNH_2_ mL^−1^, and these data therefore indicate that the gelator can take up approximately 2–3 equivalents of Ag. In general, gels composed only of calcium alginate also took up a similar amount of Ag to the gels containing DBS‐CONHNH_2_, however, as described above there was much less control over AgNP formation.

The macroscopic performance of the Ag‐loaded gels was then determined. For practical reasons, these experiments were conducted on the extended interpenetrating network hybrid gels. The thermal stability was measured as the gel‐sol transition temperature (*T*_gel_) using a simple tube inversion method (Table S3). The *T*_gel_ of the DBS‐CONHNH_2_ gel (0.4 % wt/vol) is 86 °C, but in the presence of increasing Ag loading (10 and 30 μmol of AgNO_3_ added on top of the gel), the *T*_gel_ value decreased to 37 and 30 °C respectively. This indicates that the presence of AgNPs disrupts the fibre‐fibre interactions. By contrast, the *T*_gel_ values of the DBS‐CONHNH_2_/alginate hybrid gels (0.3 % wt/vol of DBS‐CONHNH_2_ and 0.5 % wt/vol of alginate) and of the alginate gel (0.8 % wt/vol) were above 100 °C both in the absence and presence of AgNPs, indicating that the presence of the PG network increases the thermal stability of the gel, which is therefore, macroscopically not affected by the presence of AgNPs in the analysed temperature range (25–100 °C).

The mechanical properties of the Ag‐loaded gels were studied by oscillatory rheology using a parallel plate geometry (Table 1, Figure S10–S18). The DBS‐CONHNH_2_ hydrogel (0.4 % wt/vol) has an elastic modulus (*G*′) of 800 Pa, which in the presence of increasing Ag loadings (10 and 30 μmol of AgNO_3_ added on top of the gels) decreases approximately 200‐fold to around 4 Pa to give a very soft gel indeed. This supports the *T*_gel_ data and is in‐line with the view that AgNPs disrupt fibre‐fibre interactions due to the close proximity of the NPs to the gel fibres, thus reducing gel stiffness. On the other hand, the DBS‐CONHNH_2_/alginate hybrid gel (0.3 % wt/vol of DBS‐CONHNH_2_ and 0.5 % wt/vol of alginate) displayed higher *G*′ values with increasing Ag loading (*G*′ increased from 8030 to 13 800 Pa). This is probably because the presence of the PG dominates the rheological performance of the gel and therefore in this case, the AgNPs simply mechanically reinforce the gel rather than disrupting it. Indeed, a similar trend was seen when testing the calcium alginate gel alone. It is well known, in general terms, that nanoparticle additives can toughen soft materials.^27^ Overall, for the LMWG‐containing gels, as is often observed, gel stiffness is broadly inversely correlated with resistance to shear strain (as reflected in the *G*′/*G*′′ crossover point).

**Table 1:** Rheological data as determined by using oscillatory rheometry with a parallel plate geometry, for DBS‐CONHNH_2_ gels, calcium alginate gels, and interpenetrated network gels formed by the combination of the two. Loadings are given in wt/vol, and the *G*′/*G*“ crossover points (*G*′=*G*”) refer to the % shear strain at which *G*′=*G*“.

Gel	LMWG loading (wt/vol)	PG loading (wt/vol)	Ag loading [μmol]	*G*′ [Pa]	*G*′=*G*“ (shear strain)
LMWG	0.4 %	–	–	800	25.1 %
LMWG	0.4 %	–	10	3.68	39.7 %
LMWG	0.4 %	–	30	4.13	31.5 %
PG	–	0.8 %	–	2500	2.3 %
PG	–	0.8 %	10	3000	1.6 %
PG	–	0.8 %	30	6000	2.1 %
LMWG/PG	0.3 %	0.5 %	–	8030	3.8 %
LMWG/PG	0.3 %	0.5 %	10	8480	3.6 %
LMWG/PG	0.3 %	0.5 %	30	13 800	3.5 %

Most importantly, having developed and characterised AgNP‐loaded gels, we wanted to demonstrate that they display antimicrobial properties. We therefore performed a disc diffusion assay, testing the gels against two different bacterial strains: vancomycin‐resistant *Enterococcus faecium* (VRE), a Gram‐positive bacterium resistant to the antibiotic vancomycin,^28^ and *Pseudomonas aeruginosa* (PA14), a Gram‐negative bacterium.^29^ These bacterial strains are often associated with hospital‐acquired infections in immuno‐compromised individuals such as hospitalised patients in intensive care units, patients with cancer, receiving dialysis or who have had a transplant. The test was performed by placing AgNP‐loaded gels on an agar plate where the bacteria were cultured (section S8, Supporting Information). The plates were left to incubate for 24 hours and, after this time, the zones of inhibition were measured.

**Figure 5:**
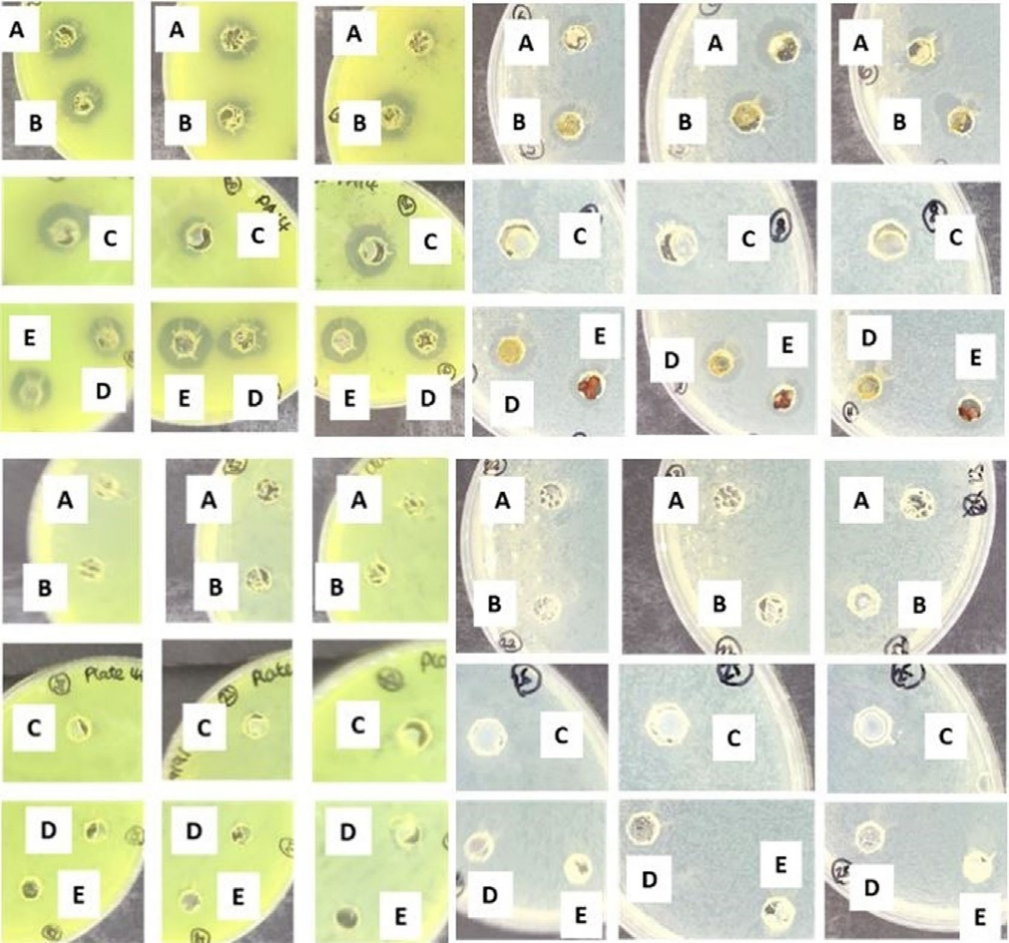
Photographic images of the disc diffusion assay. (Left) *Pseudomonas aeruginosa* (PA14). (Right) Vancomycin resistant *Enterococcus faecium* (VRE). (Top) Gels loaded with AgNPs, the dark rings indicate the zone of inhibition. (Bottom) Control gels without AgNPs. (A) Alginate gel, (B) alginate gel beads, (C) DBS‐CONHNH_2_/alginate gel, (D) DBS‐CONHNH_2_/alginate gel beads, (E) DBS‐CONHNH_2_ gel. Water, kanamycin and vancomycin controls have been reported in the Supporting Information (Figures S19 and S20).

Pleasingly, all of the gels loaded with AgNPs showed good antibacterial performance with zones of inhibition between 2 and 3 mm (Figure 5, top), comparable to previous results in the literature for AgNP‐loaded hydrogels.^20^ In contrast, gels without AgNPs showed no activity (Figure 5, bottom). Importantly, the hybrid gel beads showed similar activity to the extended interpenetrating network gels (Tables S4 and S5). These results therefore clearly demonstrate that antimicrobial performance can be achieved by the shaped hybrid gel system. These are promising preliminary results, which demonstrate that the AgNP hybrid hydrogel beads have antibacterial properties against both Gram‐positive and Gram‐negative drug‐resistant bacteria. We reason that the gel beads may be particularly appropriate for use in wound healing settings or after surgical intervention, as a result of their ease of handling.

**Figure 6:**
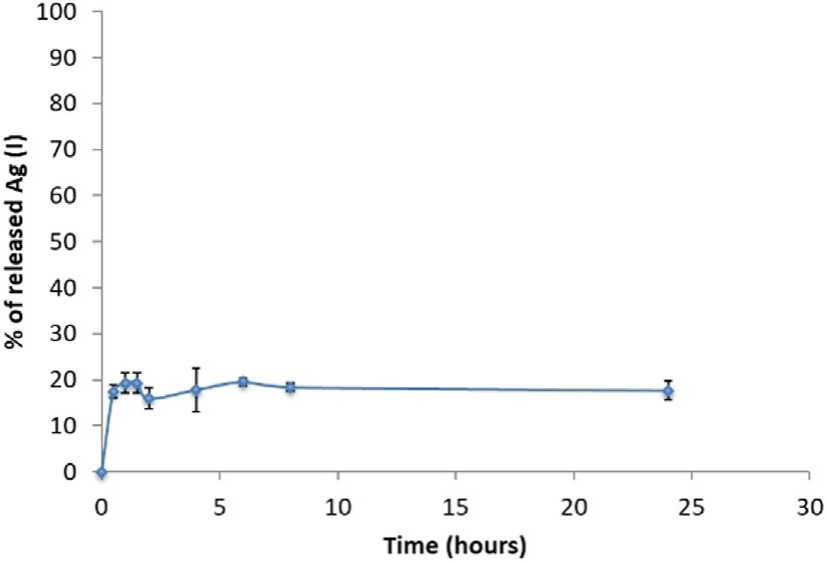
Release over time of Ag^I^ ions from the DBS‐CONHNH_2_/alginate hybrid gel beads.

There has been debate over the antimicrobial mode of action of silver nanoparticles.^30^ It has been noted that Ag^I^ ions have antimicrobial properties, but also that AgNPs have distinct mechanisms of action. We therefore determined the extent of Ag^I^ release from AgNP‐loaded gel beads in order to better understand whether silver ions were released. This was quantitatively evaluated by suspending the beads in water, and analysing the solution for Ag^I^ using a precipitation titration in the presence of NaCl and using K_2_CrO_4_ as an indicator. Studies were performed in triplicate (Tables S6 and S7). It was evident that a small amount of Ag^I^ (ca. 20 %) is initially released from the gel, but after 30 minutes no further silver ions are released (Figure 6). After 24 h, the release medium was replaced with fresh water, and no further release of silver ions was observed. This would suggest that any longer term anti‐microbial activity associated with these gel beads is more likely associated with slow AgNP release or the production of reactive oxygen species by AgNPs embedded within the gels. Further studies to understand such effects will be carried out in the future as these biomaterials are translated into a more applied setting.

## Conclusions

In conclusion, we have shown that in our two‐component DBS‐CONHNH_2_/alginate gel system that can be formulated as hybrid gel beads, both the LMWG and PG networks retains some of their key functions. The self‐assembled LMWG network remains thermally responsive within the hybrid gel beads, and is capable of generating AgNPs through in situ reduction of Ag^I^ to Ag^0^, providing excellent size control over the resulting nanoparticles. The alginate PG mechanically reinforces the gels and enables shaping of these materials into core–shell beads. In a standard DBS‐CONHNH_2_ gel, the presence of AgNPs disrupts fibre‐fibre interactions, reducing gel thermal stability and adversely affecting mechanical properties. In contrast, once calcium alginate is present in the multicomponent gel, the thermal stability remains high and gel stiffness is actually increased by AgNPs, suggesting the PG dominates the rheological performance. Once again this demonstrates the synergistic characteristics of the hybrid LMWG/PG gel. Initial screening of the AgNP‐loaded gels showed very good antimicrobial properties against drug‐resistant bacteria, in particular, VRE and *P. aeruginosa*. Furthermore, given that the individual components of the hybrid LMWG/PG gel beads are biocompatible,11c, 22 we believe that these DBS‐CONHNH_2_/alginate gels could be promising biocompatible materials.

In summary, we note that the combination of LMWG and PG reported in this paper enables:

1. Excellent control over AgNP size (because of the LMWG),
2. Improved mechanical properties (because of the PG),
3. Ability to shape the active LMWG into a core–shell bead format (because of the PG).

We suggest that these gel beads, which are very easy to handle and manipulate, and have antimicrobial properties against drug‐resistant bacteria, may be promising materials for orthopaedic applications or wound healing, particularly given that antibacterial bead‐shaped constructs are already in widespread clinical use in this setting.^21^ Further studies in this direction are in progress.

## Conflict of interest

The authors declare no conflict of interest.

## Supporting information

As a service to our authors and readers, this journal provides supporting information supplied by the authors. Such materials are peer reviewed and may be re‐organized for online delivery, but are not copy‐edited or typeset. Technical support issues arising from supporting information (other than missing files) should be addressed to the authors.

Supplementary
