# Supplementary material for: Self‐Assembled Supramolecular Hybrid Hydrogel Beads Loaded with Silver Nanoparticles for Antimicrobial Applications
Source: Chemistry. 2020 Jun 11;26(38):8452–7. doi: 10.1002/chem.202001349 (PMC7384024; doi:10.1002/chem.202001349)
Supplement: Supplementary file 1 — Supplementary [file CHEM-26-8452-s001.pdf]

Chemistry–A European Journal

Supporting Information

Self-Assembled Supramolecular Hybrid Hydrogel Beads Loaded with Silver Nanoparticles for Antimicrobial Applications

Carmen C. Piras, Clare S. Mahon, and David K. Smith*[a]

Self-Assembled Supramolecular Gel Beads Loaded with Silver Nanoparticles for Antimicrobial Applications

Carmen C. Piras,[a] Clare S. Mahon[a] and David K. Smith*[a]

a: Department of Chemistry, University of York, Heslington, York, YO10 5DD, UK

Email: david.smith@york.ac.uk

SUPPORTING INFORMATION

S1 General experimental methods

S2 Gel preparation

S3 NMR study

S4 Uptake of Ag(I)

S5 Transmission and Scanning Electron Microscopy (TEM and SEM)

S6 Thermal stability studies

S7 Rheology

S8 Disc diffusion assays

S9 Release of Ag(I) from DBS-CONHNH$_2$/alginate gel beads loaded with Ag NPs

S10 References

S1 General Experimental Methods

All compounds used in synthesis and analysis were purchased from standard commercial suppliers and used as received. The alginate employed in all the experiments was bought from Sigma Aldrich as sodium salt (2% viscosity). The synthesis of DBS-CONHNH$_2$ was performed in good yields applying previously reported methods.[1,2] TEM images were obtained on a FEI Tecnai 12 G^2 fitted with a CCD camera. Ag NPs diameters were measured using the *ImageJ* software. SEM images were taken using a JEOL JSM-7600F field emission SEM. T_{gel} values were obtained using a high precision thermoregulated oil bath using the tube inversion method and were recorded in triplicate. Rheology was measured on a Malvern Instruments Kinexus Pro+ Rheometer fitted with a 20 mm parallel plate geometry.

S2 Gel Preparation

S2.1 DBS-CONHNH$_2$ gels. DBS-CONHNH$_2$ (0.3% or 0.4% wt/vol) was suspended in water (1 mL). The suspension was sonicated to help the dispersion of the solid particles and then heated until complete dissolution of the compound. The sample was left undisturbed to cool, allowing gel formation in a few minutes.

S2.2 Alginate gels. Alginate gels were prepared by adding a $CaCl_2$ solution (5.0% wt/vol – 1 mL) to an aqueous alginate solution (0.8% wt/vol – 1 mL). Gelation occurred immediately. The excess of $CaCl_2$ solution was then removed and the gels were washed with water multiple times.

S2.3 DBS-CONHNH$_2$/alginate two-component gels (extended interpenetrating networks). DBS-CONHNH$_2$ (0.3% wt/vol in 1 mL final total volume) was suspended in water (0.5 mL) and sonicated to help the dispersion of the solid particles. An aqueous alginate solution (1.0% wt/vol - 0.5 mL) was then added. The resulting suspension was heated until complete dissolution of the DBS-CONHNH$_2$. The sample was left undisturbed for few hours to allow the formation of the DBS-CONHNH$_2$ network. A solution of $CaCl_2$ (5.0% wt/vol – 1 mL) was then added on top of each gel to crosslink the alginate chains for 30 min. The excess of $CaCl_2$ solution was then removed and the gels were washed with water multiple times.

S2.4 Alginate gel beads. Alginate gel beads were prepared by dropwise addition (20 μL/drop) of an aqueous alginate solution (0.8% wt/vol – 1 mL) to a $CaCl_2$ solution (5.0% wt/vol). The obtained beads were collected by filtration and washed with water multiple times.

S2.5 DBS-CONHNH$_2$/alginate two-component gel beads. DBS-CONHNH$_2$ (0.3% wt/vol in 1 mL final total volume) was suspended in water (0.5 mL) and sonicated to help the dispersion of the solid particles. An aqueous alginate solution (1.0% wt/vol - 0.5 mL) was subsequently added. The resulting suspension was heated until complete dissolution of the DBS-CONHNH$_2$. The hot solution was then added dropwise (20 μL/drop) to a $CaCl_2$ solution (5.0% wt/vol). The obtained beads were collected by filtration and washed with water multiple times.

S2.6 In situ formation of Ag nanoparticles into gels. Each of these gels was thoroughly washed with water multiple times and immersed in 1 or 3 mL of $AgNO_3$ solution (10 mM) for 3 days. After 3 days, the supernatant was gently removed with a pipette and the gels were washed with water multiple times. A colour change was observed in the samples in which the Ag was reduced from Ag(I) to Ag(0).

S3. NMR study

The DBS-CONHNH$_2$/alginate gel beads used for this study were prepared as described in Section S2.5 using a 0.3% wt/vol concentration of DBS-CONHNH$_2$, a 0.5% wt/vol concentration of alginate and replacing water with D_2O. Ten gel beads were isolated and transferred into a NMR tube. D_2O (0.5 mL) was then added together with DMSO (2 μL/mL), which was used as an internal standard. The sample was then placed in the spectrometer. A ^1H NMR was immediately recorded to confirm that the DBS-CONHNH$_2$ incorporated into the gel beads was in its self-assembled state. The sample was then heated to 90°C. Spectra were recorded every 10 minutes for 60 minutes and then every 30 minutes for 12.5 hours. The concentration of the mobile components was calculated by comparison of the integrals of relevant peaks (DBS-CONHNH$_2$ aromatic peaks δ = 7.55 and 7.62) to that of DMSO (δ = 2.50 ppm). The obtained data are reported in Table S1.

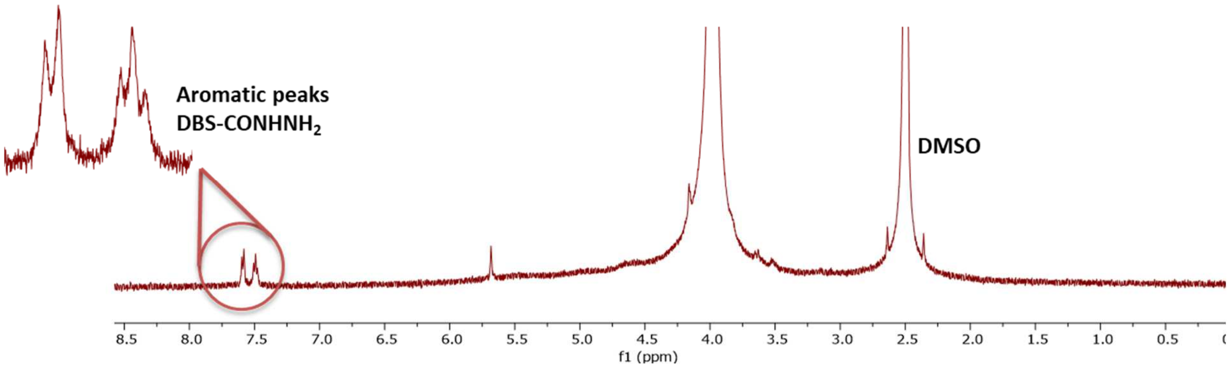

Figure S1. ^1H NMR of ten DBS-CONHNH$_2$/alginate gel beads after 1 hour at 90°C.

Table S1. Percentage of free DBS-CONHNH$_2$ into ten DBS-CONHNH$_2$/alginate gel beads over time at a constant temperature of 90°C.

Time (mins)	% of unmobilised DBS-CONHNH$_2$ into gel beads	Time (mins)	% of unmobilised DBS-CONHNH$_2$ into gel beads
0	0	360	67.70
10	41.71	390	68.66
20	46.52	420	68.34
30	48.45	450	69.62
40	55.83	480	67.38
50	56.47	510	70.26
60	57.75	540	71.23
90	58.71	570	72.19
120	59.35	600	72.83
150	58.39	630	69.30
180	59.68	660	72.51
210	59.35	690	72.19
240	58.07	720	73.79
270	60.00	750	75.72
300	65.45	780	75.72
330	69.30	810	74.44

S4 Uptake of Ag (I)

S4.1 Uptake of Ag (I) into DBS-CONHNH$_2$, alginate and DBS-CONHNH$_2$/alginate gels (extended interpenetrating networks). The gels used to estimate the uptake of Ag (I) were prepared in water

(1 mL) as described in section S2. Each of these gels was thoroughly washed with water multiple times and immersed in 1 or 3 mL of a 10 mM $AgNO_3$ solution (containing respectively 0.01 or 0.03 mmoles of Ag (I)) for 3 days (Figure S2). After 3 days, the supernatant was transferred into a vial and used to titrate a 0.05 mM solution of NaCl (2 mL) in the presence of K_2CrO_4 (5%) as an indicator. To evaluate precisely the volume of titrant used, the titration was performed by slowly adding the titrant to the NaCl solution in 10 μL drops under stirring. The volume of supernatant added as a titrant, was used to calculate the mmoles of residual Ag (I) in the supernatant (*i.e.* the Ag (I) that was not incorporated into the gel). This was subtracted from the initial mmoles of Ag (I) added, to give the mmoles of Ag (I) incorporated into the gel. To ensure data reproducibility, this experiment was performed in triplicate for each gel and average values are reported.

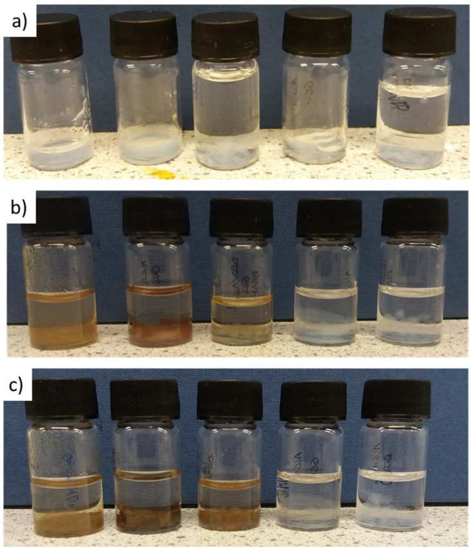

Figure S2. Comparison of colour change during Ag uptake. Gels (from left to right): DBS-$CONHNH_2$ gel, multicomponent gel extended interpenetrating networks, multicomponent gel beads, alginate gel and alginate gel beads – (a) before $AgNO_3$ addition, (b) after 24 hours, and (c) after 72 hours.

S4.2 Uptake of Ag (I) DBS-$CONHNH_2$/alginate and alginate gel beads. The gel beads used to estimate the uptake of Ag (I) were prepared as described in section S2. Each gel bead was prepared in a 20 μL volume. Since ten gel beads per type of gel were prepared, a 200 μL total volume of gel (*i.e.* 1/5 of the volume used for the gels described in Section S4.1) was considered for the subsequent addition of $AgNO_3$ in the same proportion used for the other gels. Once ready, the gel beads were thoroughly washed with water multiple times and immersed in 0.2 or 0.6 mL of a 10 mM $AgNO_3$ solution (containing respectively 0.002 or 0.006 mmoles of Ag(I)) for 3 days (Figure S3). After 3 days,

the supernatant was transferred into a vial and used to titrate a 0.05 mM solution of NaCl (2 mL) in the presence of K_2CrO_4 (5%) as an indicator. To evaluate precisely the volume of titrant used, the titration was performed by slowly adding the titrant to the NaCl solution in 10 μL drops under stirring. The volume of supernatant added as a titrant, was used to calculate the mmoles of residual Ag (I) in the supernatant (*i.e.* the Ag (I) that was not incorporated into the gel). This was subtracted to the initial mmoles of Ag (I) added, to give the mmoles of Ag (I) incorporated into the gel. To ensure data reproducibility, this experiment was performed in triplicate for each gel and average values are reported.

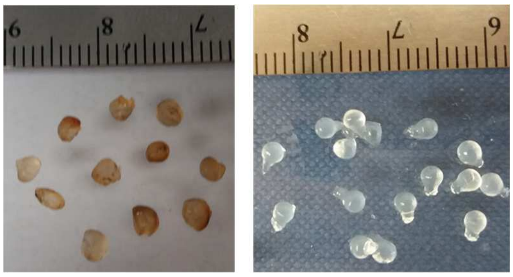

Figure S3. Comparison of colour change between (from left to right) DBS-CONHNH$_2$/alginate multicomponent gel beads and alginate gel beads after $AgNO_3$ addition (72 hours).

Table S2. Evaluation of Ag (I) uptake into DBS-CONHNH$_2$, alginate and DBS-CONHNH$_2$/alginate multicomponent gels by precipitation titration.

Gel	Loading of DBS-CONHNH$_2$ (wt/vol)	Loading of Alginate (wt/vol)	mmoles of $AgNO_3$ loaded onto gel	mmoles of Ag (I) incorporated into gel	mmoles of Ag (I) incorporated / mL of gel	% of Ag (I) incorporated
DBS-CONHNH$_2$	0.3 %	-	0.03	0.015	0.015	50 %
DBS-CONHNH$_2$	0.3 %	-	0.01	> 0.009	> 0.009	> 90 %
Alginate (EIPN)	-	0.8 %	0.03	0.015	0.015	50 %
Alginate (EIPN)	-	0.8 %	0.01	0.0057	0.0057	57 %
Alginate (10B)	-	0.8 %	0.006	0.0029	0.0145	49 %
Alginate (10B)	-	0.8 %	0.002	0.0013	0.0065	65 %
Two-component gel (EIPN)	0.3 %	0.5 %	0.03	0.014	0.014	47 %
Two-component gel (EIPN)	0.3 %	0.5 %	0.01	0.007	0.007	76 %
Two-component gel (10B)	0.3 %	0.5 %	0.006	0.0036	0.018	60 %
Two-component gel (10B)	0.3 %	0.5 %	0.002	0.0013	0.0065	65 %

*EIPN = Extended interpenetrating networks (1 mL volume/gel); 10B = 10 gel beads (20 μL volume/gel bead).

S5 Transmission and Scanning Electron Microscopy (TEM and SEM)

S5.1 Preparation of samples for TEM. Samples for TEM were obtained by placing a small amount of each sample on a copper grid. The excess sample was removed with filter paper and allowed to set for 5 min. The samples were left to rest for 30 min before taking the images. The diameter of the Ag NPs was measured on the TEM images with the *ImageJ* software on an average of 100 NPs per gel.

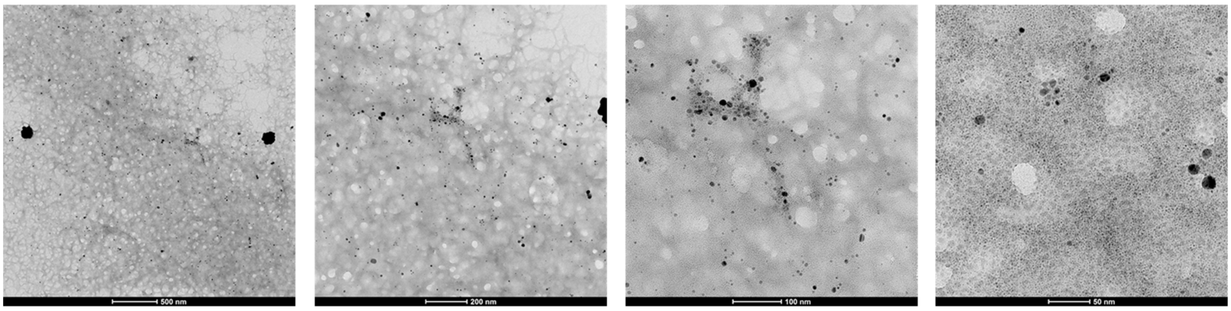

Figure S4. TEM images of DBS-CONHNH$_2$/alginate two-component gel incorporating Ag NPs (scale bars from left to right: 500, 200 100 and 50 nm).

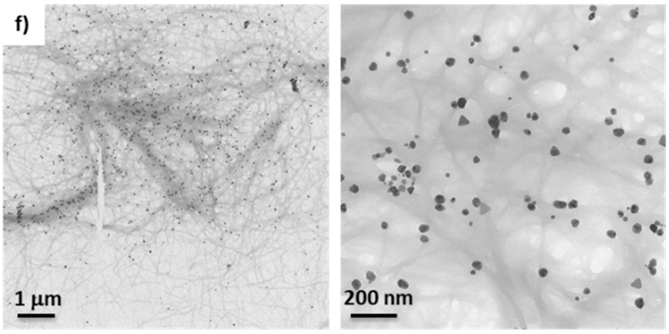

Figure S5. TEM images of DBS-CONHNH$_2$ gel incorporating Ag NPs (scale bars from left to right: 1 µm and 200 nm).

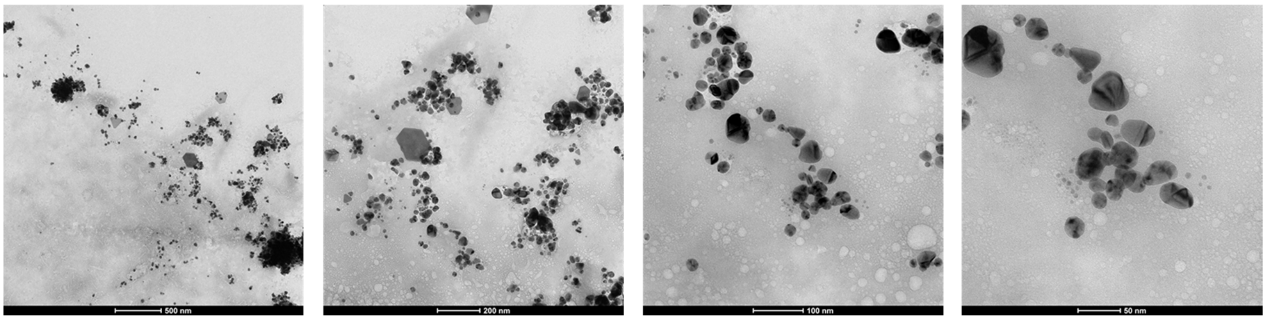

Figure S6. TEM images of alginate gel incorporating Ag NPs (scale bars from left to right: 500, 200 100 and 50 nm).

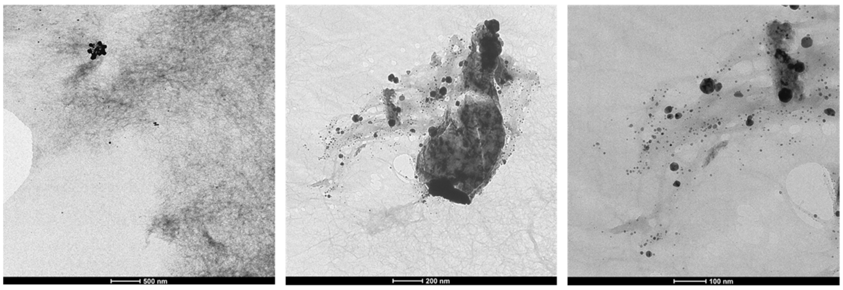

Figure S7. TEM images of alginate beads incorporating Ag NPs (scale bars from left to right: 500, 200 and 100 nm).

S5.2 Preparation of samples for SEM. Samples for SEM were obtained by freeze drying the gels on copper shim pieces. The freeze-dried samples were then mounted on stubs and the images recorded.

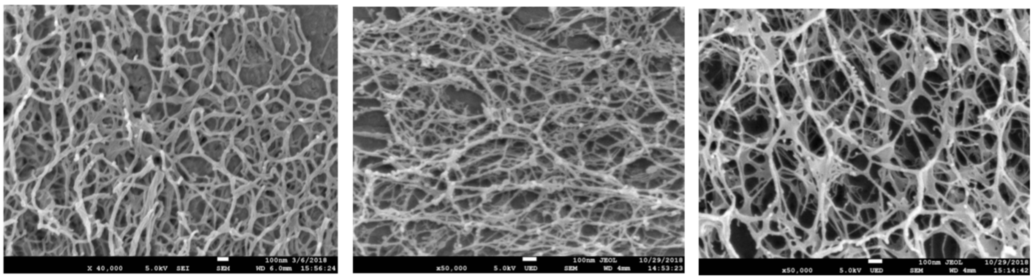

Figure S8. SEM images of (from left to right) DBS-CONHNH$_2$ gel), DBS-CONHNH$_2$/alginate two-component gel interpenetrating networks and alginate gel (scale bars: 100 nm).

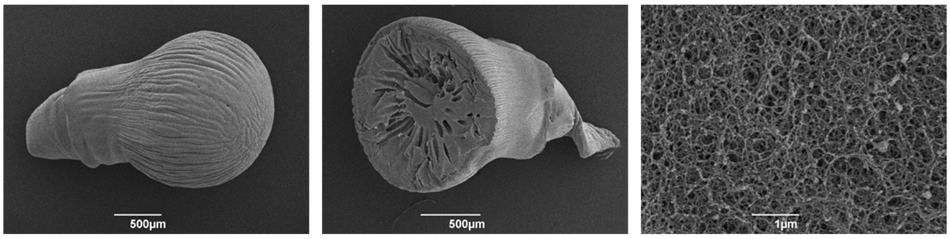

Figure S9. SEM images of an alginate gel bead (left) and its cross-section (middle and right incorporating Ag NPs (scale bars from left to right: 500, 500 and 1 µm).

S6 Thermal stability studies

S6.1 T_{gel} Values. All the gels for T_{gel} determination were prepared as described in Section S2 and S3 in 7 mL vials (diameter: 2 cm, height: 5 cm). The gels were placed in a high precision thermo-

regulated oil bath with an initial temperature of 25°C. The temperature was increased by 1°C/min until 100°C. Every minute the gels were checked by tube inversion method and T_{gel} was considered as the temperature at which the gel began to run down the sides of the vial. These experiments were performed in triplicate to ensure reproducibility and the average is reported. Errors are estimated at ±2°C.

Table S3. T_{gel} values of gels formed by individual gelators and the DBS-CONHNH$_2$/alginate two-component gel (interpenetrating networks) with and without Ag NPs.

Gel (1 mL total volume)	Loading of DBS-CONHNH$_2$ (wt/vol)	Loading of Alginate (wt/vol)	mmoles of $AgNO_3$ loaded onto gel	T_{gel}
DBS-CONHNH$_2$	0.3%	-	-	86 °C
DBS-CONHNH$_2$	0.3%	-	0.01	37 °C
DBS-CONHNH$_2$	0.3%	-	0.03	30 °C
Alginate	-	0.8%	-	>100 °C
Alginate	-	0.8%	0.01	>100 °C
Alginate	-	0.8%	0.03	>100 °C
DBS-CONHNH$_2$/alginate two-component gel	0.3%	0.5%	-	>100 °C
DBS-CONHNH$_2$/alginate two-component gel	0.3%	0.5%	0.01	>100 °C
DBS-CONHNH$_2$/alginate two-component gel	0.3%	0.5%	0.03	>100 °C

S7 Rheology

S7.1 Sample preparation. Gel samples for rheology were prepared as described in Section S2 using bottomless vials as templates to obtain the intended gel dimensions. The measurements were carried out at 25°C using a 20 mm parallel plate and a gap of 2 mm. To avoid solvent evaporation and keep the sample hydrated, a solvent trap was used, and the internal atmosphere was kept saturated. Amplitude sweep experiments were performed in the range of 0.05-100% strain at a 1 Hz frequency to identify the linear viscoelastic region. Frequency sweep experiments were performed between 0.1 and 100 Hz using a shear strain of 0.25%. The measurements were repeated three times to ensure reproducibility and the average data are shown.

S7.2 Rheological data collected

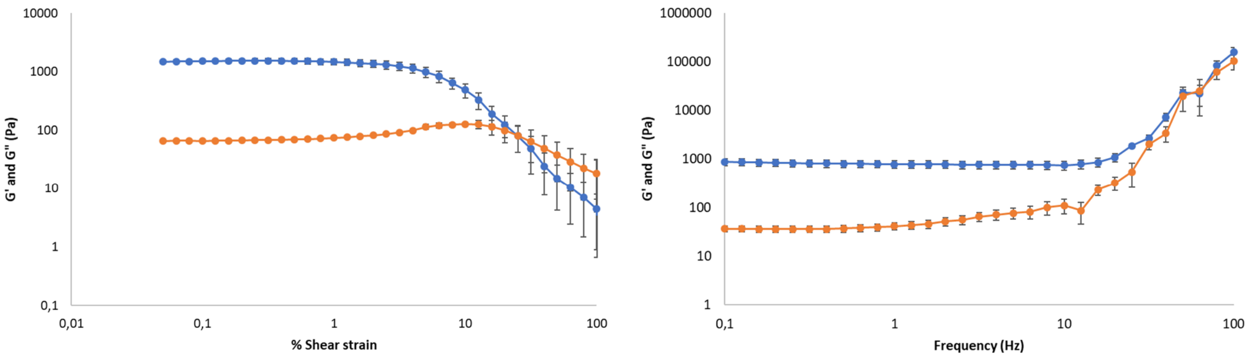

Figure S10. Elastic (G', blue circles) and viscous (G", orange circles) moduli of DBS-CONHNH$_2$ hydrogel (0.4% wt/vol) with increasing shear strain (left) and frequency (right).

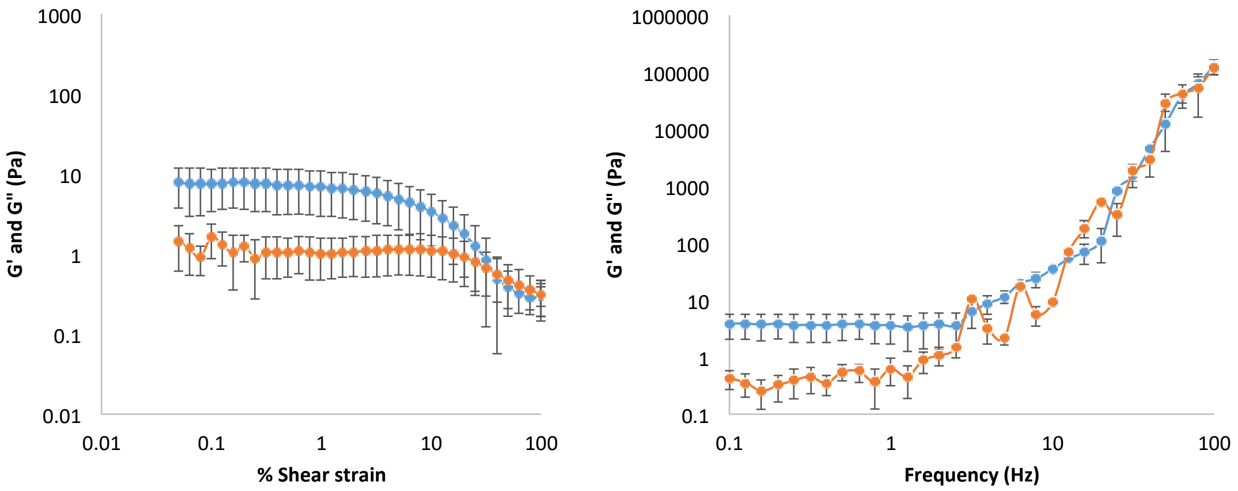

Figure S11. Elastic (G', blue circles) and viscous (G", orange circles) moduli of DBS-CONHNH$_2$ hydrogel (0.4% wt/vol - loaded with 1 mL $AgNO_3$ 10 mM) with increasing shear strain (left) and frequency (right).

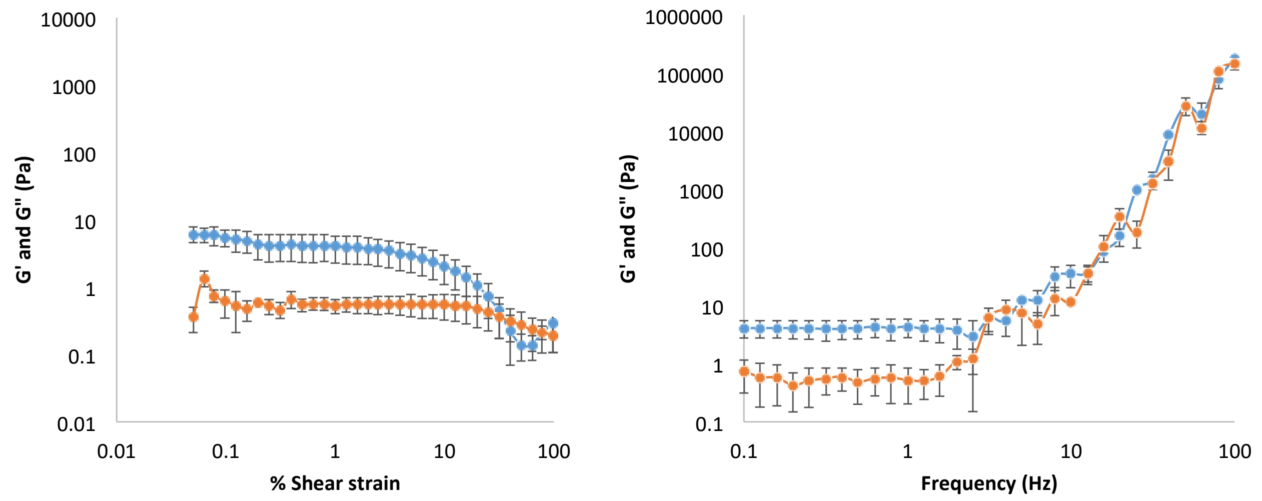

Figure S12. Elastic (G’, blue circles) and viscous (G”, orange circles) moduli of DBS-CONHNH$_2$ hydrogel (0.4% wt/vol - loaded with 3 mL $AgNO_3$, 10 mM) with increasing shear strain (left) and frequency (right).

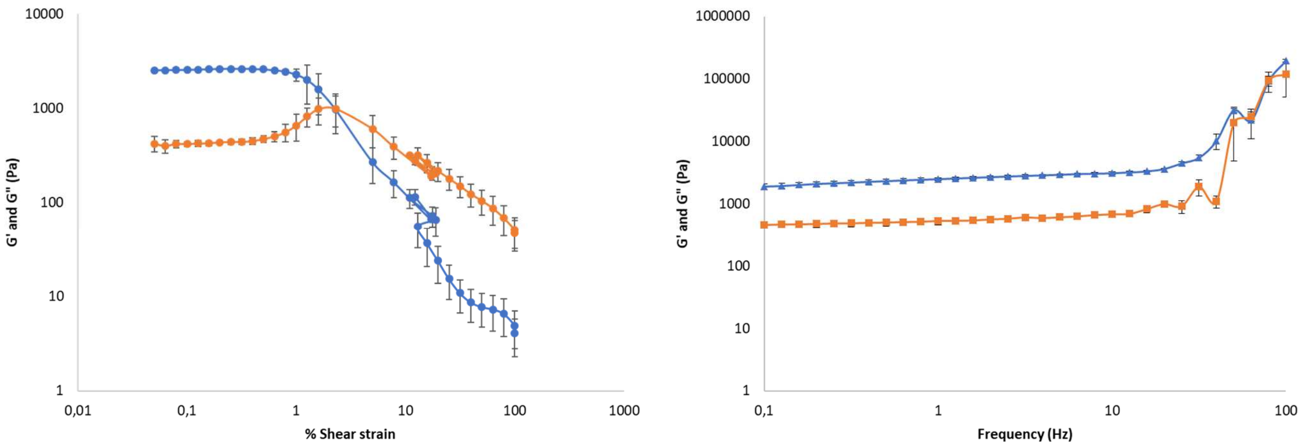

Figure S13. Elastic (G’, blue circles) and viscous (G”, orange circles) moduli of alginate hydrogel (0.8% wt/vol) with increasing shear strain (left) and frequency (right).

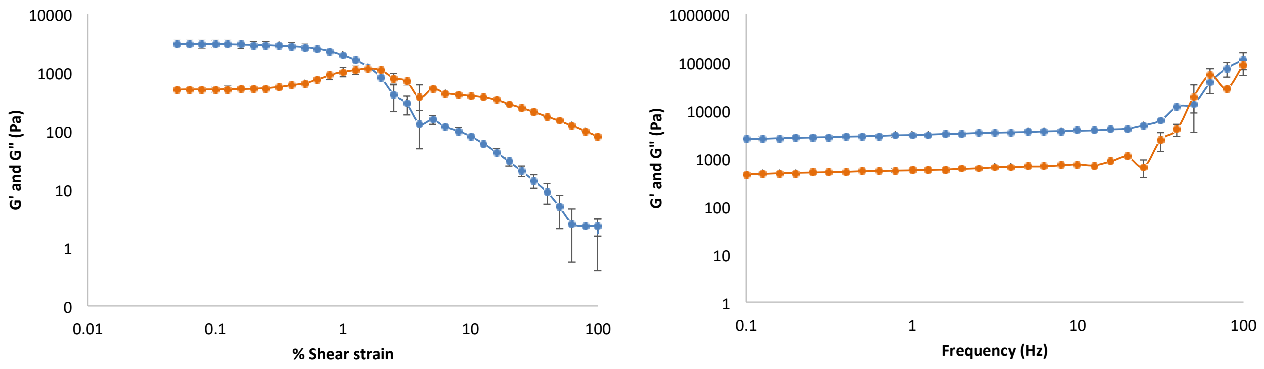

Figure S14. Elastic (G’, blue circles) and viscous (G”, orange circles) moduli of alginate hydrogel (0.8% wt/vol- loaded with 1 mL $AgNO_3$, 10 mM) with increasing shear strain (left) and frequency (right).

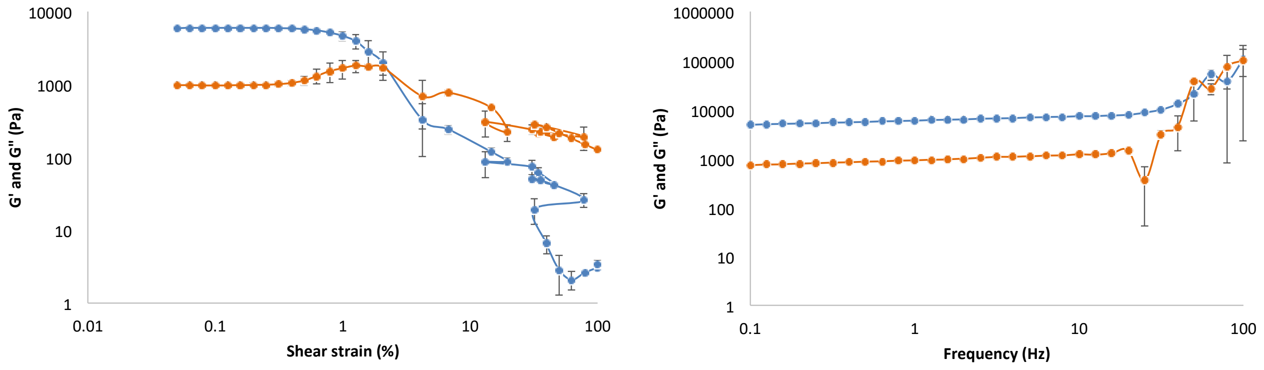

Figure S15. Elastic (G’, blue circles) and viscous (G”, orange circles) moduli of alginate hydrogel (0.8% wt/vol- loaded with 3 mL $AgNO_3$ 10 mM) with increasing shear strain (left) and frequency (right).

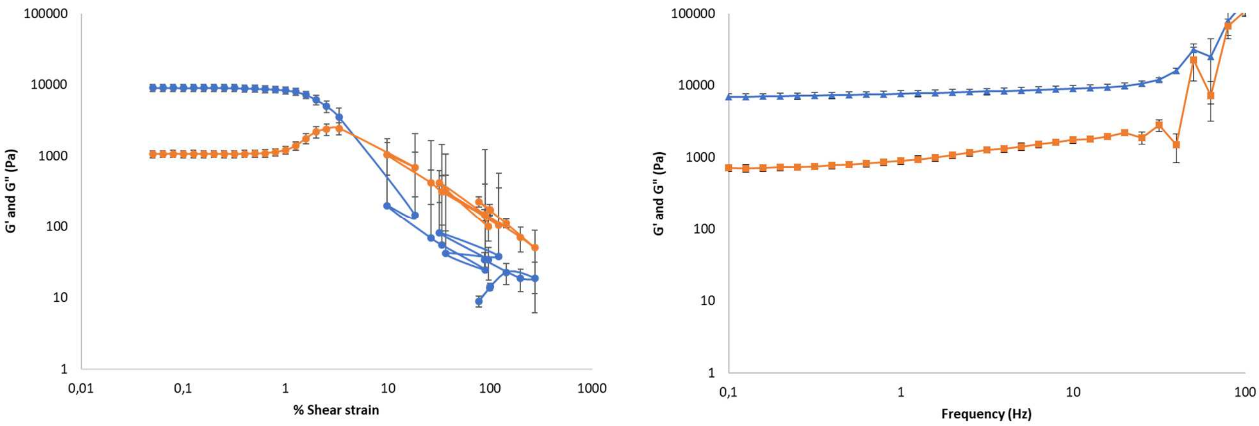

Figure S16. Elastic (G’, blue circles) and viscous (G”, orange circles) moduli of DBS-CONHNH$_2$/alginate multicomponent hydrogel (0.3% wt/vol DBS-CONHNH$_2$ and 0.5% wt/vol alginate) with increasing shear strain (left) and frequency (right).

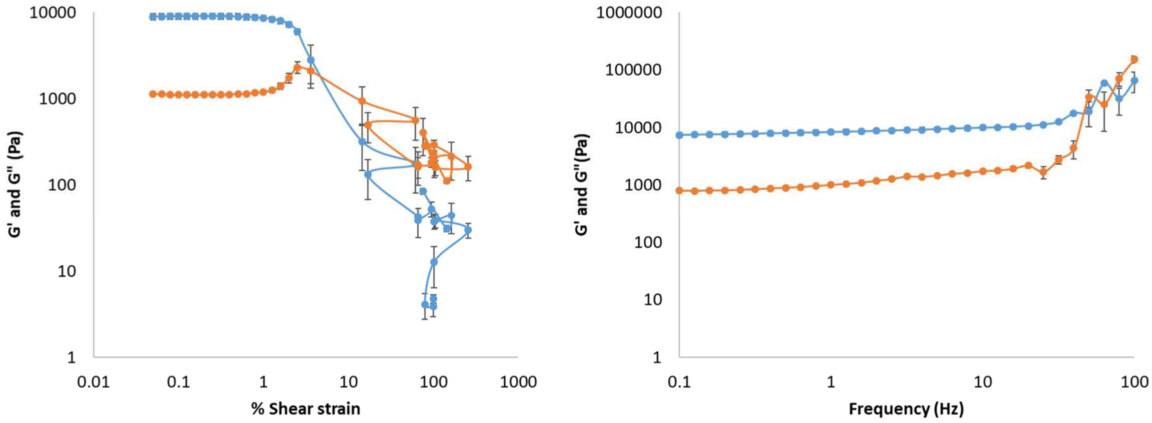

Figure S17. Elastic (G’, blue circles) and viscous (G”, orange circles) moduli of DBS-CONHNH$_2$/alginate multicomponent hydrogel (0.3% wt/vol DBS-CONHNH$_2$ and 0.5% wt/vol alginate - loaded with 1 mL $AgNO_3$, 10 mM) with increasing shear strain (left) and frequency (right).

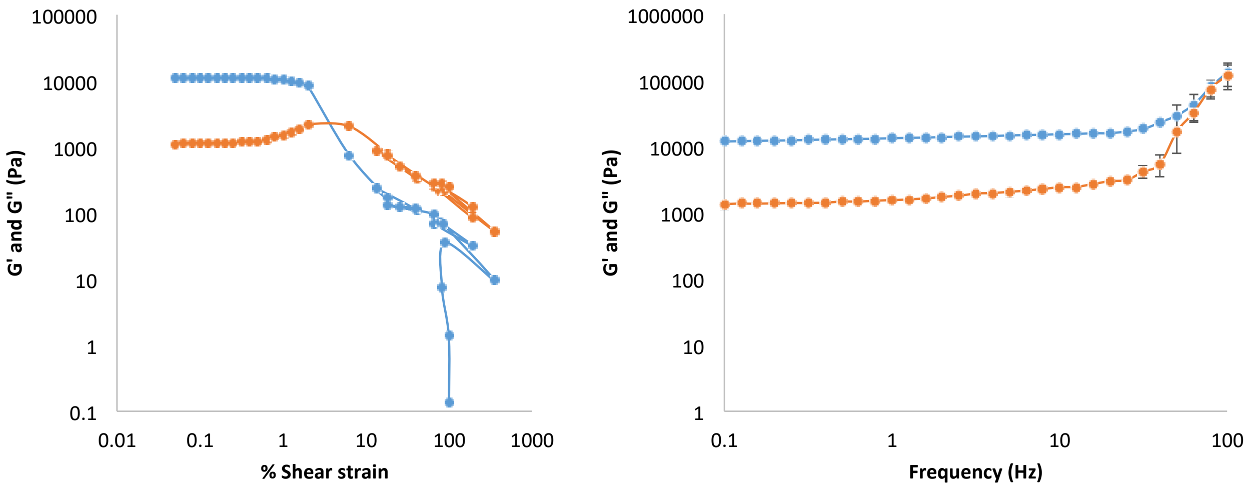

Figure S18. Elastic (G', blue circles) and viscous (G", orange circles) moduli of DBS-$CONHNH_2$/alginate multicomponent hydrogel (0.3% wt/vol DBS-$CONHNH_2$ and 0.5% wt/vol alginate - loaded with 3 mL $AgNO_3$, 10 mM) with increasing shear strain (left) and frequency (right).

S8 Disc diffusion assay

S8.1 Gel preparation. The gels were prepared in sterile conditions in 75 μL volume in a 96 well plate as described in Section S2. The gel beads were prepared using 20 μL volume/gel bead and 4 beads were placed in each well. The *in situ* formation of Ag NPs was induced by immersing the gels in 75 μL of a 10 mM solution of $AgNO_3$ for 3 days. After 3 days, the supernatant was gently removed and the gels were carefully washed with water multiple times. The gels were then transferred on the agar plates, where the bacteria were cultured, for the disc diffusion assay.

S8.2 Disc diffusion assay. Cells from glycerol stocks were used to innoculate LB medium (5 mL; 10 g L^{-1} tryptone, 5 g L^{-1} yeast, 10 g L^{-1} NaCl), performed in triplcate for each strain. Cultures were grown at 37 °C with shaking at 180 rpm for 17 h. Cultures were diluted to OD600 0.1, followed by a further 1:100 dilution, with autoclaved water. These cultures were spread onto LB agar (75 μL per plate; 10 g L^{-1} tryptone, 5 g L^{-1} yeast, 10 g L^{-1} NaCl, 15 g L^{-1} agar). A 200 μL pipette tip was used to remove discs from the agar, into which gels were inserted. Plates were incubated at 37°C for 24 h before inhibition radii were measured. Bacterial Strains: vancomycin-resistant *Enterococcus faecium* (VRE):

DSM 17050 (https://www.dsmz.de/collection/catalogue/details/culture/dsm-17050. *Pseudomonas aeruginosa* (PA14)

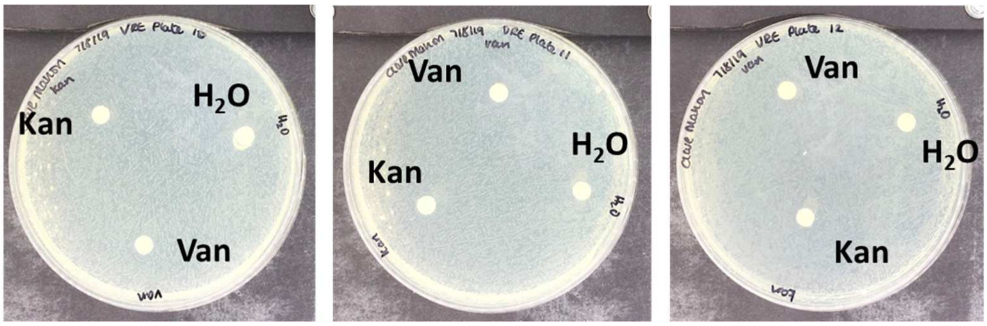

Figure S19. Disc diffusion assay - vancomycin-resistant *E. faecium* (VRE) (VRE). Controls: water (H_2O), kanamycin (Kan), vancomycin (Van).

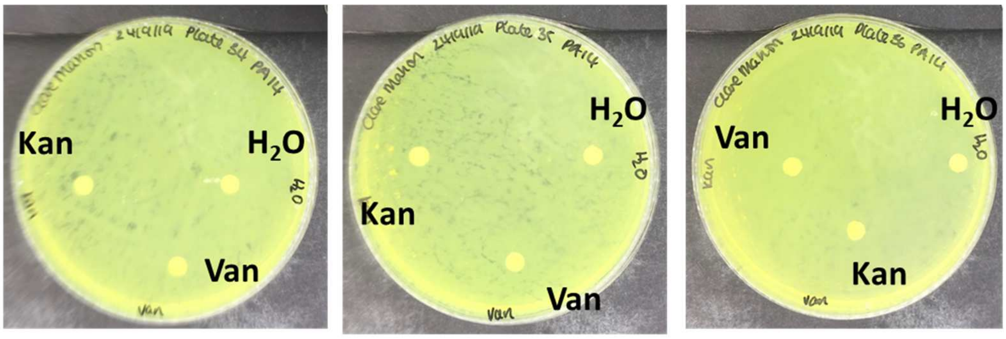

Figure S20. Disc diffusion assay – *P. aeruginosa* (PA14). Controls: water (H_2O), kanamycin (Kan), vancomycin (Van).

Table S4. Inhibition Radii from disc diffusion assay - vancomycin-resistant *E. faecium* (VRE).

Vancomycin-resistant *E. faecium* (VRE)					
Gel	Inhibition radii - Replicate 1 (mm)	Inhibition radii - Replicate 2 (mm)	Inhibition radii - Replicate 3 (mm)	Inhibition radii - Mean (mm)	Standard Error
Alginate (discs)	2	2	3	2.33	0.33
Alginate (beads)	3	3	3	3.00	0.00
Alginate (discs) – no Ag NPs	0	0	0	0.00	0.00
Alginate (beads) – no Ag NPs	0	0	0	0.00	0.00
Alginate/DBS-CONHNH$_2$ (discs)	3	1	2	2.00	0.58
Alginate/DBS-CONHNH$_2$ (beads)	2	2	3	2.33	0.33
Alginate/DBS-CONHNH$_2$ (discs) – no Ag NPs	0	0	0	0.00	0.00
Alginate/DBS-CONHNH$_2$ (beads) – no Ag NPs	0	0	0	0.00	0.00
DBS-CONHNH$_2$	3	3	3	3.00	0.00
DBS-CONHNH$_2$– no Ag NPs	0	0	0	0.00	0.00

Table S5. Inhibition Radii from disc diffusion assay - *Pseudomonas aeruginosa* (PA14).

Pseudomonas aeruginosa (PA14)					
Gel	Inhibition radii - Replicate 1 (mm)	Inhibition radii - Replicate 2 (mm)	Inhibition radii - Replicate 3 (mm)	Inhibition radii - Mean (mm)	Standard Error
Alginate (discs)	4	2	3	3.00	0.58
Alginate (beads)	4	3	1	2.67	0.88
Alginate (discs) – no Ag NPs	0	0	0	0.00	0.00
Alginate (beads) – no Ag NPs	0	0	0	0.00	0.00
Alginate/DBS-CONHNH$_2$ (discs)	4	2	3	3.00	0.58
Alginate/DBS-CONHNH$_2$ (beads)	3	2	3	2.67	0.33
Alginate/DBS-CONHNH$_2$ (discs) – no Ag NPs	0	0	0	0.00	0.00
Alginate/DBS-CONHNH$_2$ (beads) – no Ag NPs	0	0	0	0.00	0.00
DBS-CONHNH$_2$	4	2	3	3.67	0.33
DBS-CONHNH$_2$– no Ag NPs	0	0	0	0.00	0.00

S9 Release of Ag(I) from DBS-CONHNH$_2$/alginate gel beads loaded with Ag NPs

S9.1 Preparation of gel beads. The DBS-CONHNH$_2$/alginate gel beads (40 beads/sample) were prepared as described in section S2 using 20 μL volume/gel bead and washed with water multiple

times. The *in situ* formation of Ag NPs was induced by immersing the gels in 3 ml of a 10 mM solution of $AgNO_3$ for 24 hours. After 24 hours, the supernatant was removed and used to calculate the exact amount of Ag (I) incorporated by precipitation titration as described in Section S4.2. To ensure reproducibility, three samples of 40 beads each were prepared for each time point and the exact amount of Ag (I) incorporated in each sample was calculated and reported in Table S6.

Table S6. Amount of Ag(I) incorporated in each sample of 40 gel beads used for the release study.

**Sample name**	**Mmoles of Ag (I) incorporated into 40 gel beads**	**% of Ag (I) incorporated**
A	0.013	44.4
B	0.014	47.4
C	0.016	54.5
D	0.012	41.2
E	0.016	52.4
F	0.013	44.4
G	0.013	44.4
H	0.015	50.0
I	0.014	47.4
J	0.016	54.5
K	0.016	54.5
L	0.014	47.4
M	0.017	58.3
N	0.016	54.5
O	0.012	41.2
P	0.012	41.2
Q	0.013	44.4
R	0.015	50.0
S	0.014	47.4
T	0.013	44.4
U	0.014	47.4
V	0.014	47.4
W	0.013	44.4
X	0.016	54.5

S9.2 Release of Ag(I) from DBS-CONHNH$_2$/alginate gel beads. Each sample (40 gel beads/sample) was immersed in 2 ml of water. At the specified time intervals, the release medium was removed and used to titrate a 0.1 mM solution of NaCl (1 mL) in the presence of K_2CrO_4 (5% - 1 mL) as an indicator. To evaluate precisely the volume of titrant used, the titration was performed by slowly adding the titrant to the NaCl solution in 10 µL drops under stirring. The volume of supernatant added as a titrant, was used to calculate the mmoles of released Ag (I) (Table S7). To ensure data

reproducibility, this experiment was performed in triplicate for each gel and average values were reported in the final graph.

Table S7. Ag(I) released from each sample.

Sample name	Time point (hours)	mmoles of Ag (I) released from gel beads	% of Ag (I) released	Average %	Standard error
A	0.5	0.0025	18.75	17.51	1.38
B	0.5	0.0026	18.04		
C	0.5	0.0026	16.08		
D	1	0.0027	21.59	19.32	2.20
E	1	0.0027	17.20		
F	1	0.0026	19.74		
G	1.5	0.0028	21.43	19.31	2.19
H	1.5	0.0026	17.09		
I	1.5	0.0028	19.82		
J	2	0.0028	17.46	15.98	2.28
K	2	0.0022	13.58		
L	2	0.0025	17.59		
M	4	0.0026	14.65	17.86	4.76
N	4	0.0028	16.97		
O	4	0.0029	23.81		
P	6	0.0025	20.24	19.57	0.78
Q	6	0.0027	20.00		
R	6	0.0028	18.78		
S	8	0.0025	17.59	18.42	0.82
T	8	0.0026	19.23		
U	8	0.0026	18.52		
V	24	0.0025	17.37	17.67	1.99
W	24	0.0027	20.00		
X	24	0.0026	16.08		

S10 References

[1] B. O. Okesola and D. K. Smith, *Chem. Commun.*, 2013, **49**, 11164-11166.

[2] D. J. Cornwell, B. O. Okesola and D. K. Smith, *Soft Matter*, 2013, **9**, 8730-8736.
